# Supplementary material for: Mid-old cells are a potential target for anti-aging interventions in the elderly
Source: Nat Commun. 2023 Nov 22;14:7619. doi: 10.1038/s41467-023-43491-w (PMC10665435; doi:10.1038/s41467-023-43491-w)
Supplement: Supplementary file 1 — Supplementary Information [file 41467_2023_43491_MOESM1_ESM.pdf]

Supplementary Table 1. Patient information of colon donors used in this study.

	Young (n=18)	Old (n=20)	p value
**Sex: Male (%)**	9 (50.0)	12 (60.0)	0.38[a]
**Age: Median [range]**	33.5 [20-40]	82.0 [80-90]	<0.0001[b]
**Diagnosis (%)**			0.0003[a]
**Diverticulum-related diseases**	9 (50.0)	0 (0.0)	
**Tumor**	9 (50.0)	20 (100.0)	

[a]*p* values are obtained using Chi-square test. [b]*p* value is obtained using two-tailed Student's *t* test.

Supplementary Table 2. Patient information of lung donors used in this study.

	Young (n=20)	Old (n=34)	p value
**Sex: Male (%)**	14 (70.0)	19 (55.9)	0.30[a]
**Age: Median [range]**	27 [19-38]	77 [75-83]	<0.0001[b]
**Diagnosis (%)**			<0.0001[a]
**Sequestration**	1 (5.0)	0 (0.0)	
**Bulla**	1 (5.0)	0 (0.0)	
**Pneumothorax**	13 (65.0)	0 (0.0)	
**Tumor**	5 (25.0)	34 (100.0)	

[a]*p* values are obtained using Chi-square test. [b]*p* value is obtained using two-tailed Student's *t* test.

Supplementary Table 3. Patient information of liver donors used in this study.

	Young (n=19)	Old (n=30)	p value
**Sex: Male (%)**	12 (63.2)	14 (46.7)	0.26[a]
**Age: Median [range]**	29 [20-42]	83 [80-87]	<0.0001[b]
**Diagnosis (%)**			<0.0001[a]
**Liver transplant donor**	19 (100.0)	0 (0.0)	
**Tumor**	0 (0.0)	28 (93.4)	
**Bile duct stone**	0 (0.0)	1 (3.3)	
**Fatty change**	0 (0.0)	1 (3.3)	

[a]*p* values are obtained using Chi-square test. [b]*p* value is obtained using two-tailed Student's *t* test.

Supplementary Table 4. Patient information of skin donors used in this study.

	Young (n=31)	Old (n=7)	p value
**Sex: Male (%)**	14 (45.2)	3 (42.9)	0.91[a]
**Age: Median [range]**	19 [3-39]	72 [71-81]	<0.0001[b]
**Diagnosis (%)**			0.29[a]
**Morphea**	3 (9.7)	1 (14.3)	
**Idiopathic Guttate Hypomelanosis**	12 (38.7)	4 (57.1)	
**Linchen striatus**	1 (3.2)	0 (0.0)	
**Nevus**	4 (12.9)	0 (0.0)	
**Bilateral telangiectatic macule**	1 (3.2)	0 (0.0)	
**Atrophoderma**	1 (3.2)	0 (0.0)	
**Lentiginosis**	1 (3.2)	0 (0.0)	
**Confluent and reticulated Papillomatosis**	6 (19.4)	0 (0.0)	
**Acanthosis nigricans**	1 (3.2)	0 (0.0)	
**Café au lait macule**	1 (3.2)	0 (0.0)	
**Livedoid vasculitis**	0 (0.0)	1 (14.3)	
**Steroid-induced atrophy**	0 (0.0)	1 (14.3)	

[a]*p* values are obtained using Chi-square test. [b]*p* value is obtained using two-tailed Student's *t* test.

Supplementary Table 5. Patient information of serum donors used in this study.

	Young (n=10)	Old (n=10)	p value
**Sex: Male (%)**	5 (50.0)	8 (80.0)	0.16[a]
**Age: Median [range]**	38.5 [30-40]	75 [71-86]	< 0.0001[b]
**Diagnosis (%)**			1.00[a]
**Tumor**	10 (100.0)	10 (100.0)	

[a]*p* values are obtained using Chi-square test. [b]*p* value is obtained using two-tailed Student's *t* test.

Supplementary Table 6. Real-time PCR primer sequences used in this study.

Gene name	Forward Primer	Reverse Primer
**Human**		
**p53 (*TP53*)**	GCC CAA CAA CAC CAG CTC CT	CCT GGG CAT CCT TGA GTT CC
**p21^{Waf1} (*CDKN1A*)**	ATT AGC AGC GGA ACA AGG AGT CAG ACA T	CTG TGA AAG ACA CAG AAC AGT ACA GGG T
**p16^{INK4A}(*CDKN2A*)**	CTC GTG CTG ATG CTA CTG AGG A	GGT CGG CGC AGT TGG GCT CC
IL6	AAT TCG GTA CAT CCT CGA CGG	GGT TGT TTT CTG CCA GTG CC
IL1B	CTG TCC TGC GTG TTG AAA GA	TTG GGT AAT TTT TGG GAT CTA CA
**IL8 (*CXCL8*)**	CTG GCC GTG GCT CTC TTG	CCT TGG CAA AAC TGC ACC TT
**MIP1α (*CCL3*)**	TGTTGCCAAACAGCCACAC	CAGAGCAAACAATCACAAACACAC
**MIP3a (*CCL20*)**	ATGTGCTGTACCAAGAGTTT	TTACATGTTCTTGACTTTTT
**MCP2 (*CCL8*)**	TATCCAGAGGCTGGAGAGCTAC	TGGAATCCCTGACCCATCTCTC
CCL2	AGC AGC AAG TGT CCC AAA GA	TTG GGT TTG CTT GTC CAG GT
CCL5	TCC CAC AGG TAC CAT GAA GGT C	GCA ATG TAG GCA AAG CAG CAG
CXCL1	CTT GCC TCA ATC CTG CAT C	CCT TCT GGT CAG TTG GAT TTG
SAA1	TTT CTG CTC CTT GGT CCT GG	CTC TGG CAT CGG TGA TCA CT
SLIT2	GTC AAT GAC CTA CTG GCC TCA TTC	TGA TTG CTA CAC ACC ATT TCG TTT C
CXCL12	TGC CAG AGC CAA CGT CAA G	CAG CCG GGC TAC AAT CTG AA
**IL1rα (*IL1RN*)**	ATG GAG GGA AGA TGT GCC TGT C	GTC CTG CTT TCT GTT CTC GCT C
IL11	GGA CCA CAA CCT GGA TTC CCT G	AGT AGG TCC GCT CGC AGC CTT
IL18BP	GTG TCC AGC ATT GGA AGT GAC C	GGA GGT GCT CAA TGA AGG AAC C
**Caveolin1 (*CAV1*)**	CCA AGG AGA TCG ACC TGG TCA A	GCC GTC AAA ACT GTG TGT CCC T
**Caveolin2 (*CAV2*)**	TTC TCT TTG CCA CCC TCA GCT G	GAA GCA TCG TCC TAC GCT CGT A
KPNA2	CTG TTG GCT CTC CTT GCA GTT C	GCA GGA TTC TTG TTG CGG CAA AG
KPNB1	CTG CTT CCT GAA GCT GCC ATC A	CTT CAG CCA GAC TGG AGA AAG C
MMP1	AAG CGT GTG ACA GTA AGC TA	AAC CGG ACT TCA TCT CTG
MMP3	GGA CAA AGG ATA CAA CAG GGA CCA	GAA CCG AGT CAG GTC TGT GAG TG
MMP9	CAC GAC GTC TTC CAG TAC CGA GA	CAT AGG TCA CGT AGC CCA CTT GGT
FBXO30	ACA AAT GGA GAC TGT GTG GCA TC	GCC ATT AGG CAA AGC ACT GGA TG
FBXO32	CAC TGG TCC AAA GAG TCG GCA A	GCA CAA AGG CAG GTC AGT GAA G
TRIM63	AAG CCA GTG GTC ATC TTG CCG T	CTC CAG ACA TGG ACA CTG AGC T
COL4A1	TGT TGA CGG CTT ACC TGG AGA C	GGT AGA CCA ACT CCA GGC TCT C
COL4A2	GGA TAA CAG GCG TGA CTG GAG T	CTT TGC CAC CAG GCA GTC CAA T
COL4A3	GGA CAA AGG AGA ACC AGG TCT C	AGT GCT GCC CAA ATC TCC TCT G
COL3A1	TGG TCT GCA AGG AAT GCC TGG A	TCT TTC CCT GGG ACA CCA TCA G
COL1A1	GAT TCC CTG GAC CTA AAG GTG C	AGC CTC TCC ATC TTT GCC AGC A
AQP3	CCG TGA CCT TTG CCA TGT GCT T	TTG TCG GCG AAG TGC CAG ATT G
NHE2	AAG GAA GGT CAC GTC CAG TG	TGT CTC TCA CTT GTG TCG GC
NHE3	TCT CGG CCA TCG AGG ACA TA	ACA TTC AGG ATC CGG TCT CG
ATP1A1	GGC AGT GTT TCA GGC TAA CCA G	TCT CCT TCA CGG AAC CAC AGC A
SCNN1A	GTG CCT ACA TCT TCT ATC CGC G	GTC TGA GGA GAA GTC AAC CTG G
SFTPD	AAG TGG GCT TCC AGA TGT TG	CTG TGC CTC CGT AAA TGG TT

SOX2	AGG GCC GGA CAG CGA ACT G	TTT GCA CCC CTC CCA TTT C
OCT4	CCT GAA GCA GAA GAG GAT CAC C	AAA GCG GCA GAT GGT CGT TTG G
TNF	CTC TTC TGC CTG CTG CAC TTT G	ATG GGC TAC AGG CTT GTC ACT C
SBLC	CACACATCTACTCTCTTACAGTTCTAT	TTATCTGGGAGGACCAATGTAATC
SALRNA1	TGCATGTGTGTGTGTGTGTG	CTCTGGGAATCTGGAACCAA
ROR	GCC TGA GAG TTG GCA TGA AT	AAA ACC TCA CTC CCA TGT GC
MYH2	GAC AGC CAA GAA GAG GAA ACT GG	ACC TGC CAT CTC TTC TGT GAG G
MYBPC1	CCT ATG AGG TCC GCA TCT TTG C	GTC GTG TCA GTG ACA GAG TCC A
ROBO1	GGC CCC ACT CCC CCT GTT CG	TCC TCT TCT GGC GCA TCC GTA TCC
ROBO2	GTT TGT GTT GCG AGG AAC TAT CT	GTT TTG TCG GAA GTC ATC TCG TA
ROBO3	CAG TGT CCG ATG GAA GAA GG	GTC CAT CTC CTG CAC ATT GG
ROBO4	GAC ACT TGG CGT TCC ACC TC	AGA GCA AGG AGC GAC GAC AG
IL1R1	GTG CTT TGG TAC AGG GAT TCC TG	CAC AGT CAG AGG TAG ACC CTT C
IL1R2	GGC TAT TAC CGC TGT GTC CTG A	GAG AAG CTG ATA TGG TCT TGA GG
IL6R	GAC TGT GCA CTT GCT GGT GGA T	ACT TCC TCA CCA AGA GCA CAG C
IL17D	TGA GCA GGC GCG CAA CGC GA	GCA GTA GGC TTC AGG CAG GTA C
IL6ST	CAC CCT GTA TCA CAG ACT GGC A	TTC AGG GCT TCC TGG TCC ATC A
IL10	TCTCCGAGATGCCTTCAGCAGA	TCAGACAAGGCTTGGCAACCCA
IL4	CCG TAA CAG ACA TCT TTG CTG CC	GAG TGT CCT TCT CAT GGT GGC T
IL13	ACG GTC ATT GCT CTC ACT TGC C	CTG TCA GGT TGA TGC TCC ATA CC
SH2B3	CCT GAT GCT CAT GGA GTG TTC C	GGA AAG TGG AGG TGC TGC ACA C
PADI2	GAT GAG CAG CAA GCG AAT CAC C	GCT CCT TCT TGA GGA TGT CAC G
RNF113A	CGA CTT GAG CAG CGA AGA GGA A	CGC TCT TTC TCT GTG TCC AGC T
**TGFβ (*TGFB1*)**	CCC AGC ATC TGC AAA GCT C	GTC AAT GTA CAG CTG CCG CA
**β-Actin (*ACTB*)**	CCC TGG CAC CCA GCA C	GCC GAT CCA CAC GGA GTA C
**Mouse**		
Nhe3	TCT GTT TGT CAG CAC CAC TCT C	TCA CGA TGC TCG CTC CTC TTC
Scnn1a	TGA TGG TGG CTT CAA CGT GAG G	AGT GCA GTC TCC GTA GTT GCC T
**β-Actin (*Actb*)**	TTG AAC ATG GCA TTG TTA CCA ACT	TCA AAC ATG ATC TGG GTC ATC TTT

Supplementary Table 7. The shRNA sequences used in this study.

Gene	Target Sequence
**sh-SLIT2**	#1; ACTAGAGAGACTGCGTTTAAA
	#2; TGAACCTTCTCTCCCTATATG
**sh-p21^{Waf1}**	#1; CGCTCTACATCTTCTGCCTTA
	#2; GACAGATTTCTACCACTCCAA
**sh-SAA1**	#1; CTGACATGAGAGAAGCCAATT
	#2; CATCGGCTCAGACAAATACTT
**sh-MMP9**	#1; GATGCGTGGAGAGTCGAAATC
	#2; TGCATAAGGACGACGTGAATG

Supplementary Figure 1

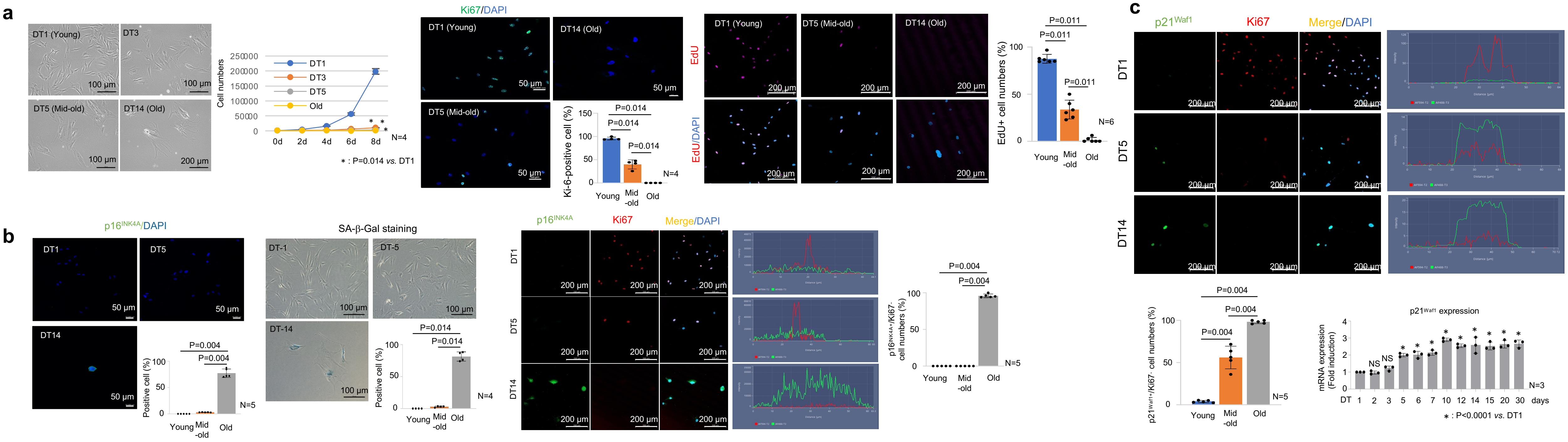

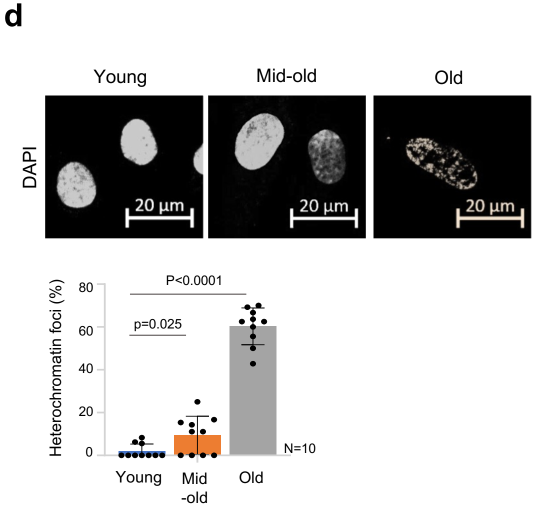

Supplementary Figure 1. The expression of senescence-related genes in young, mid-old, and old fibroblasts. a The left panel displays representative morphology images of young (DT1), mid-old (DT5), and senescent (old) human primary fibroblasts (DT14). The proliferation capacity of the cells was assessed using the cell growth rate for 8 days (see a graph of the left panel). Ki67 (green) ICC analysis was performed for young, mid-old, and old cells, respectively (middle panel). The right panel shows the EdU incorporation assay of young, mid-old, and old human primary fibroblasts. **b** Two senescence markers, p16^{INK4A} and SA-β-Gal, along with the proliferation marker Ki67, were examined in the young, mid-old, and old human primary fibroblasts. Immunocytochemistry (ICC) analysis for p16^{INK4A} (left panel) and SA-β-Gal (middle panel) were performed. ICC for p16^{INK4A} (green) and Ki67 (red) was conducted in young, mid-old, and old cells (right panel). The graph on the right represents the measured fluorescence intensity of representative cell. The percentage of p16^{INK4A+}/Ki67^{-} cells is presented as a bar graph. **c** Double IF staining for p21^{Waf1} (green) and Ki67 (red) was performed in young, mid-old, and old fibroblasts (upper panel). The percentage of p21^{Waf1+}/Ki67^{-} cells is displayed as the dot plot (lower left panel). The graph on the right represents the measured fluorescence intensity of representative cells. The mRNA level of p21^{Waf1} in a cDNA microarray (GSE41714) conducted during replicative senescence is presented as a bar graph (lower right panel). **d** Heterochromatin foci stained by DAPI is shown in young (DT1), mid-old (DT5), and old (DT17), respectively (upper panel). Related quantification data is shown in the lower panel. The *p* values in **a-b, c (lower left panel)** and **d** were determined using the one-tailed Mann-Whitney U test. The *p* value in **c (lower right panel)** was determined using the two-tailed Student's *t* test. All graphs are presented as mean ± SD.

Supplementary Figure 2

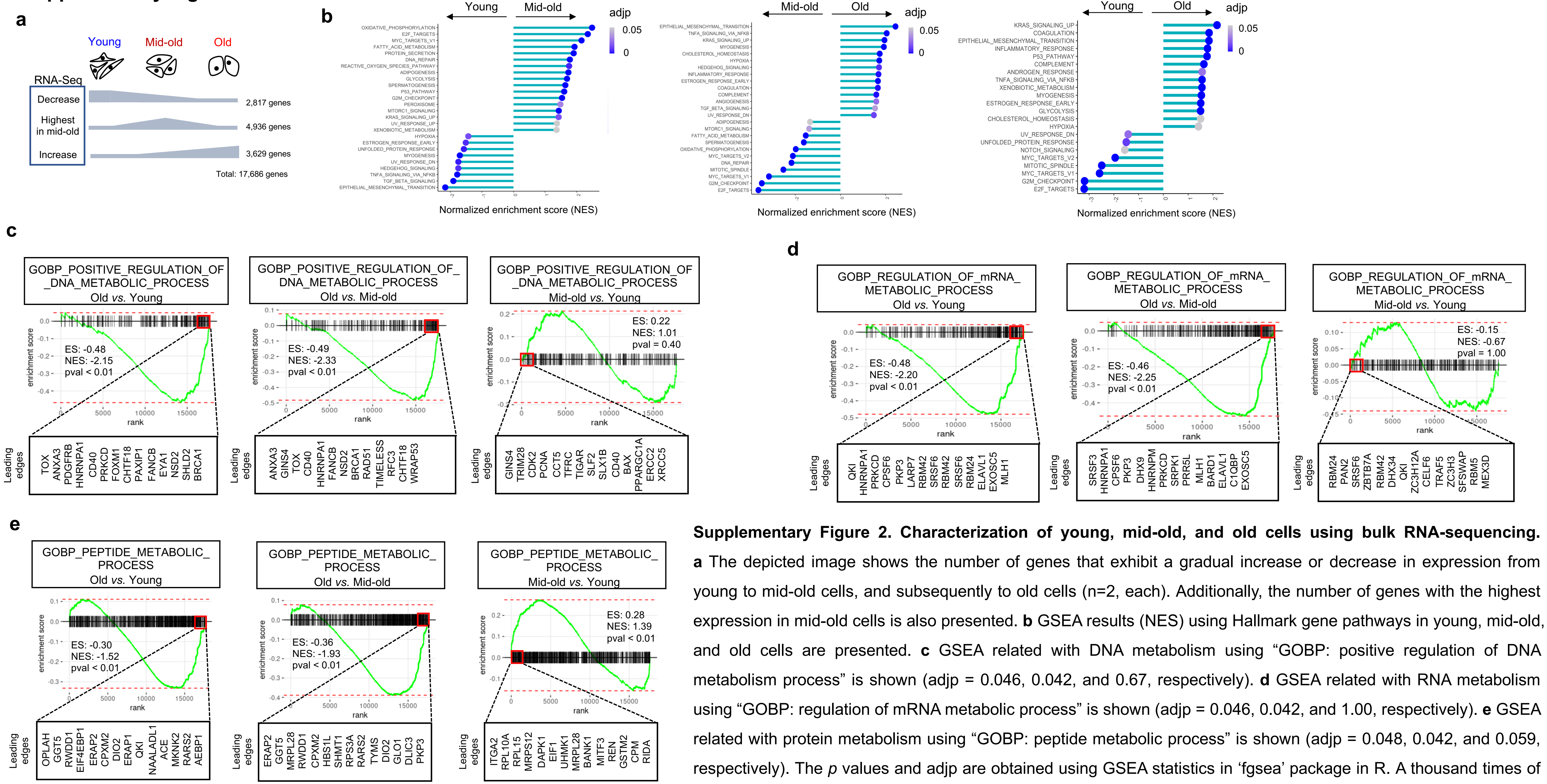

Supplementary Figure 2. Characterization of young, mid-old, and old cells using bulk RNA-sequencing. **a** The depicted image shows the number of genes that exhibit a gradual increase or decrease in expression from young to mid-old cells, and subsequently to old cells (n=2, each). Additionally, the number of genes with the highest expression in mid-old cells is also presented. **b** GSEA results (NES) using Hallmark gene pathways in young, mid-old, and old cells are presented. **c** GSEA related with DNA metabolism using “GOBP: positive regulation of DNA metabolism process” is shown (adjp = 0.046, 0.042, and 0.67, respectively). **d** GSEA related with RNA metabolism using “GOBP: regulation of mRNA metabolic process” is shown (adjp = 0.046, 0.042, and 1.00, respectively). **e** GSEA related with protein metabolism using “GOBP: peptide metabolic process” is shown (adjp = 0.048, 0.042, and 0.059, respectively). The *p* values and adjp are obtained using GSEA statistics in ‘fgsea’ package in R. A thousand times of permutation were performed in **b-e** for adjustment. ES: enrichment score; NES: normalized enrichment score; adjp: adjusted *p* value.

Supplementary Figure 3

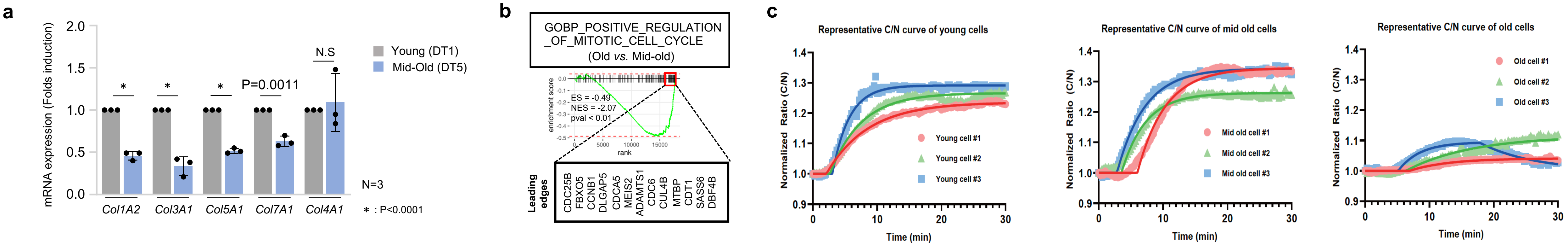

Supplementary Figure 3. Functional characteristics of young, mid-old, and old cells. a Collagen (*COL1A2*, *COL3A1*, *COL5A1*, *COL7A1*, and *COL4A1*) mRNA levels in young and mid-old fibroblasts are shown (cDNA microarray, GSE41714). Data are presented as mean ± SD. The *p* value was determined using two-tailed Student's *t* test. **b** GSEA of old *vs.* mid-old using "GOBP: positive regulation of mitotic cell cycle" are shown (n=2, each, adjp = 0.042). A thousand times of permutation were performed for adjustment. The *p* value is obtained using GSEA statistics in 'fgsea' package in R. ES: enrichment score; NES: normalized enrichment score; adjp: adjusted *p* value. **c** Representative Erk1/2-KTR-mClover translocation C/N curves for young (left panel), mid-old (middle panel), and old cells (right panel) are shown (n=3, each). Fluorescent images were acquired every 15 sec for 30 mins with 100 msec exposure time after serum (20%) stimulation.

Supplementary Figure 4

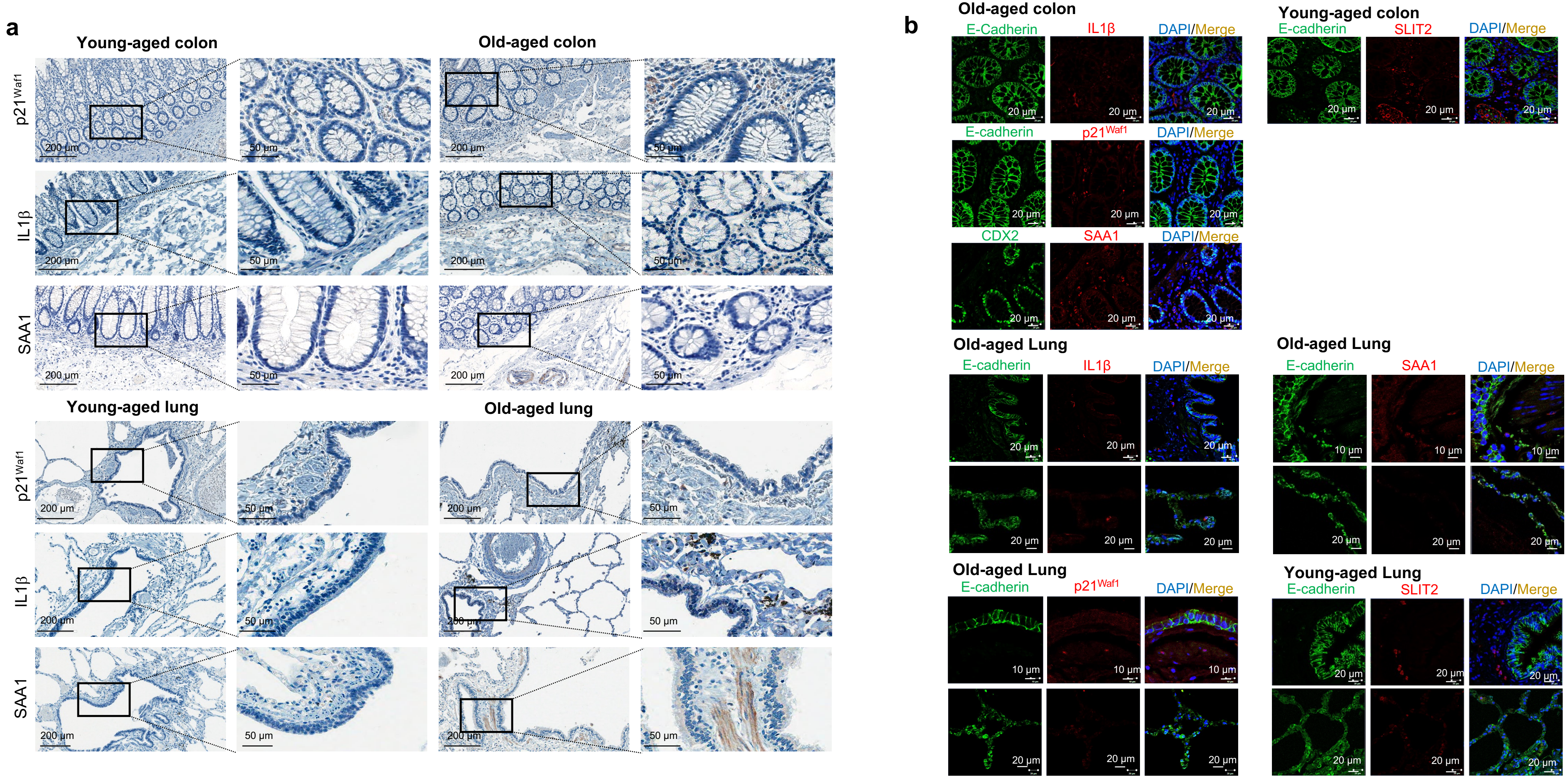

Supplementary Figure 4. The expression of mid-old-specific markers in colon and lung epithelial cells. a IHC of human young and old aged colon and lung tissues using $p21^{Waf1}$, IL1β and SAA1 antibodies were performed. **b** IF staining was performed using various combinations of antibodies in tissues obtained from elderly and young subjects. The staining combinations included E-cadherin (green)/IL1β (red), E-cadherin (green)/$p21^{Waf1}$ (red), E-cadherin (green)/SAA1 (red), CDX2 (green)/SAA1 (red), and E-cadherin (green)/SLIT2 (red). DAPI (blue) was used to visualize nuclei.

Supplementary Figure 5

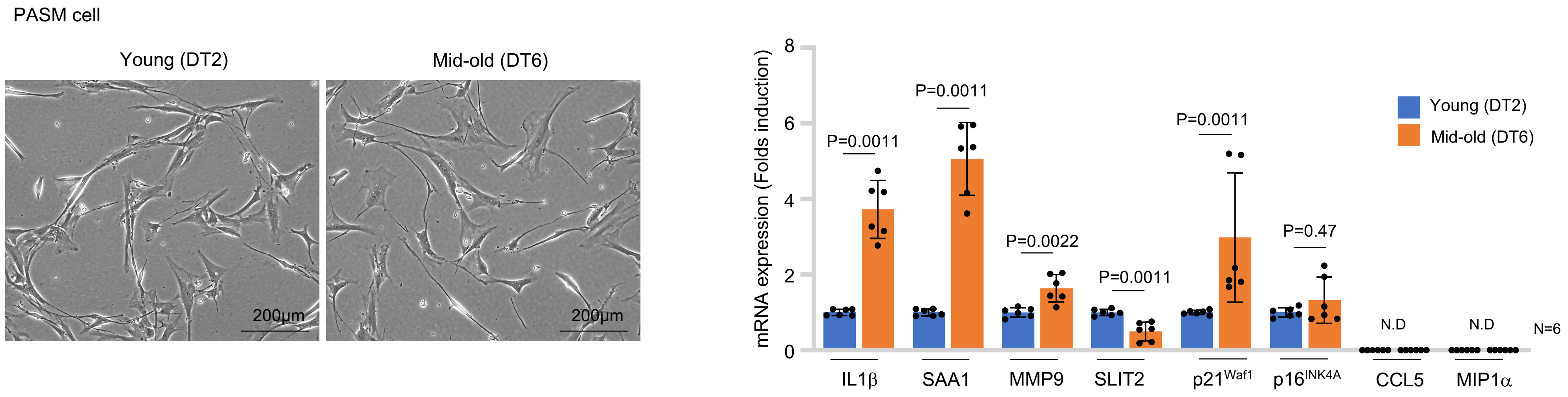

Supplementary Figure 5. The expression of mid-old-specific markers in primary vascular smooth muscle cells *in vitro*. The left panel shows the morphology of primary pulmonary artery smooth muscle cells (PASM) serially sub-cultured (young PASM (DT2) and mid-old PASM (DT6)). The right panel displays the mRNA expression of IL1β, SAA1, MMP9, SLIT2 $p21^{Waf1}$, $p16^{INK4A}$, CCL5, and MIP1α. The *p* value was determined using the one-tailed Mann-Whitney U test. Data are presented as mean ± SD. 'N.D' is not detected.

Supplementary Figure 6

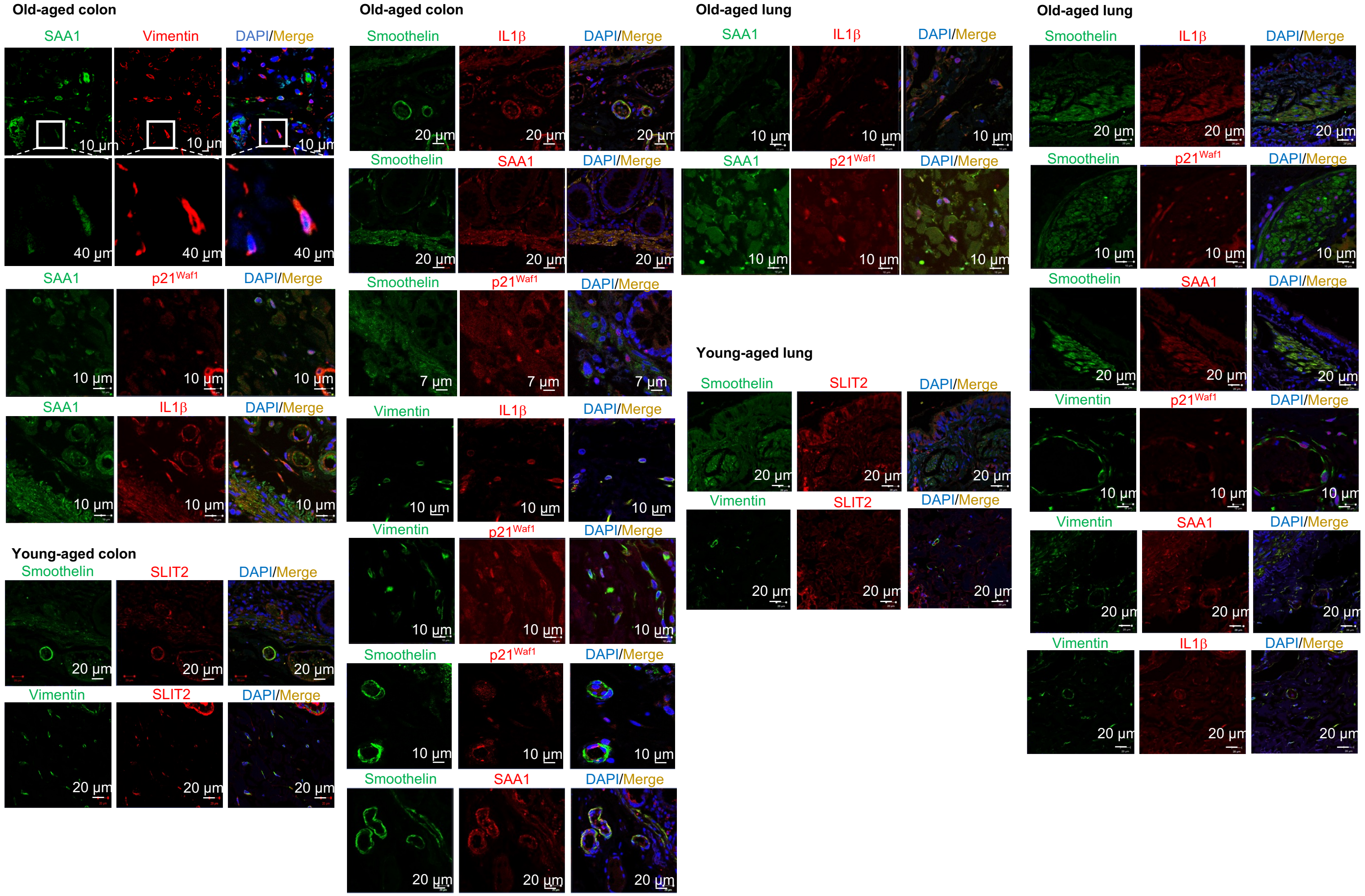

Supplementary Figure 6. p21^{Waf1}, SAA1, IL1β and SLIT2 expression in human tissues. IF staining was performed using various combinations of antibodies in tissues obtained from elderly and young subjects. The staining combinations included SAA1 (green)/vimentin (red), SAA1 (green)/p21^{Waf1} (red), SAA1 (green)/IL1β (red), smoothelin (green)/IL1β (red), smoothelin (green)/SAA1 (red), smoothelin (green)/p21^{Waf1} (red), vimentin (green)/p21^{Waf1} (red), vimentin (green)/SAA1 (red), vimentin (green)/IL1β (red), smoothelin (green)/SLIT2 (red), and vimentin (green)/SLIT2 (red). DAPI (blue) was used to label nuclei.

Supplementary Figure 7

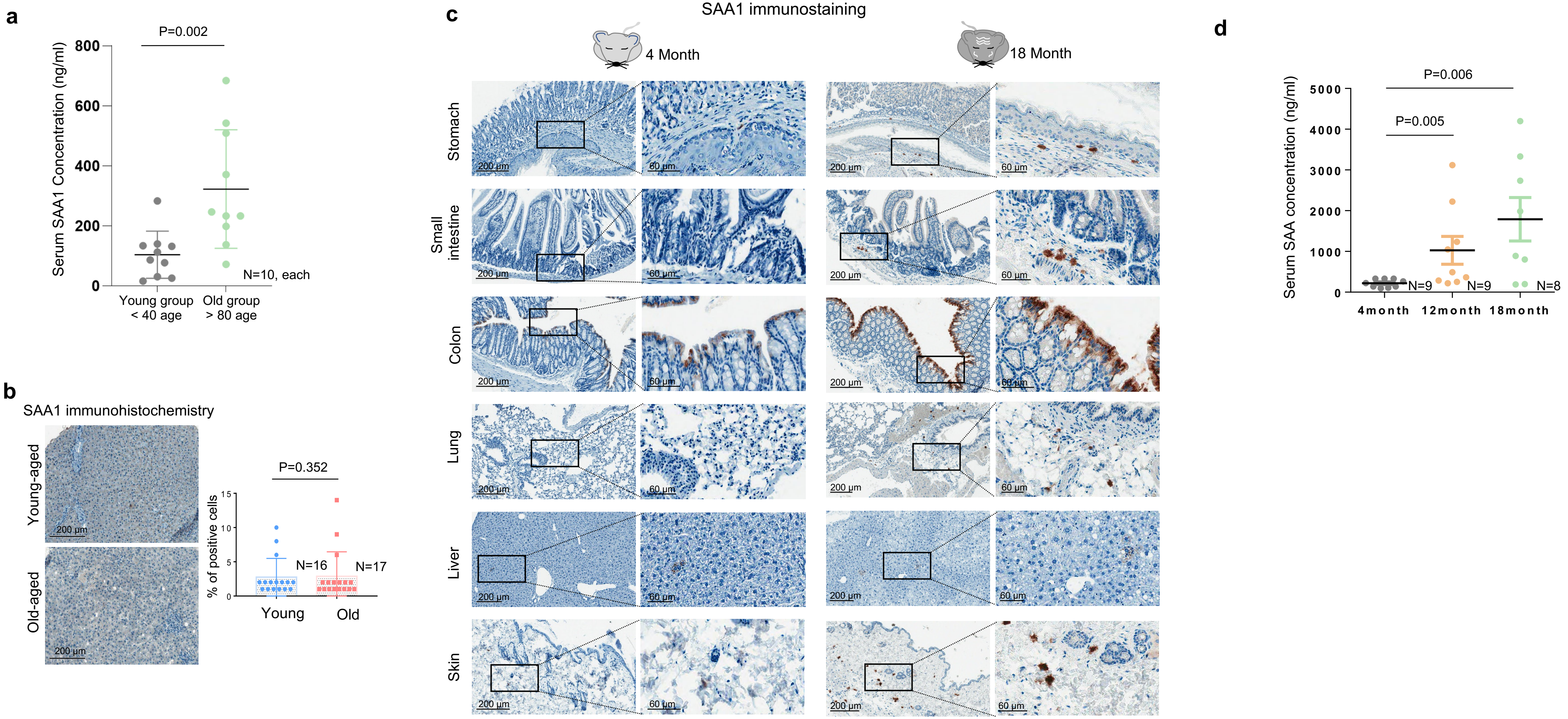

Supplementary Figure 7. SAA1 expression in old-aged individuals and mice. a Determination of SAA1 concentration in human serum from patients with benign or malignant diseases. The *p* value was calculated using the two-tailed Student's *t* test. **b** IHC analysis of human liver tissue using SAA1 antibody was performed. SAA1 expression status in hepatocytes was presented as the percentage of positive cells. The *p* value was determined using the one-tailed Mann-Whitney U test. **c** IHC analysis of SAA1 in the specified tissues from young (4-month-old) and old (18-month-old) male mice. **d** Mouse serum SAA1 was measured in 4-, 12-, and 18-month-old male and female mice using an ELISA. The *p* value was calculated using the one-tailed Mann-Whitney U test. All graphs are presented as mean ± SD.

Supplementary Figure 8

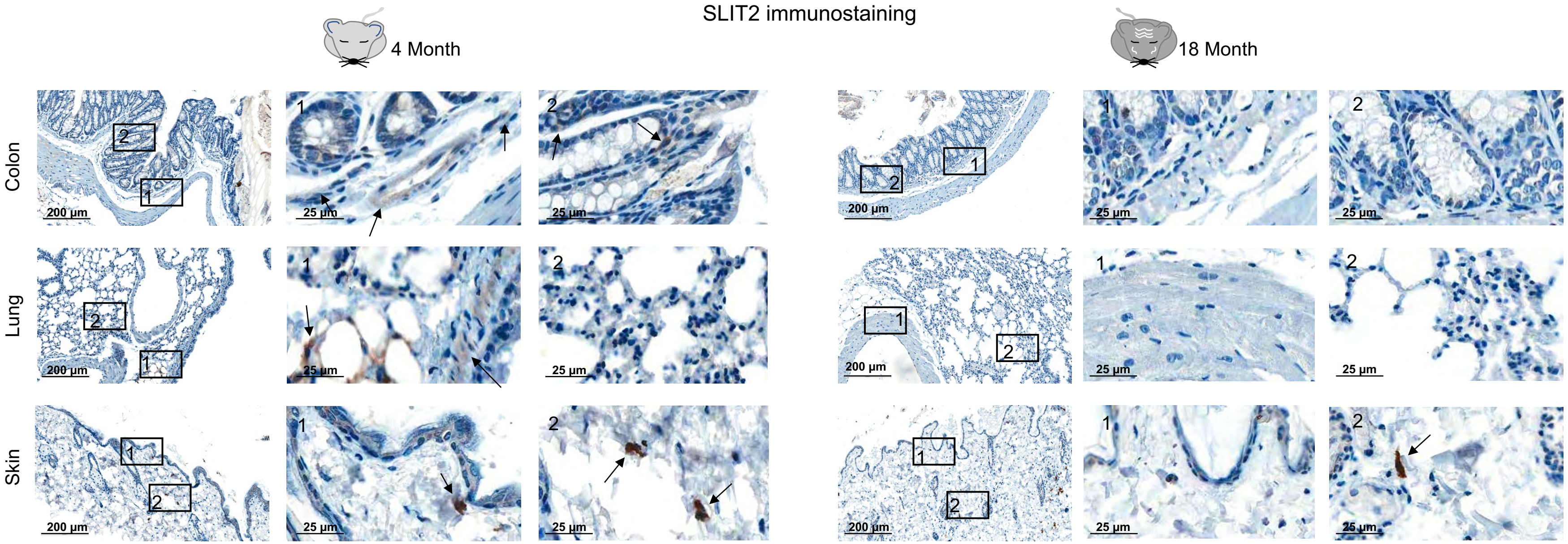

Supplementary Figure 8. SLIT2 expression in old-aged mouse tissues. IHC analysis of SLIT2 in the specified tissues from young (4-month-old) and old (18-month-old) male mice. Arrow indicates SLIT2-positive cells.

Supplementary Figure 9

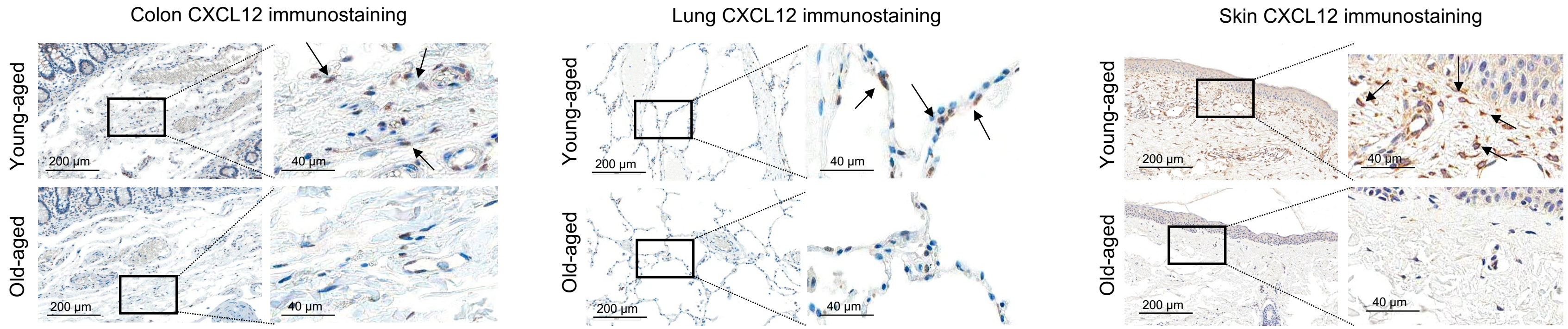

Supplementary Figure 9. CXCL12 expression in tissue from elderly subjects. IHC analysis was conducted to examine the expression of CXCL12 in the stromal region of colon, lung, and skin tissues obtained from elderly and young subjects. Arrows indicate the CXCL12-positive cells.

Supplementary Figure 10

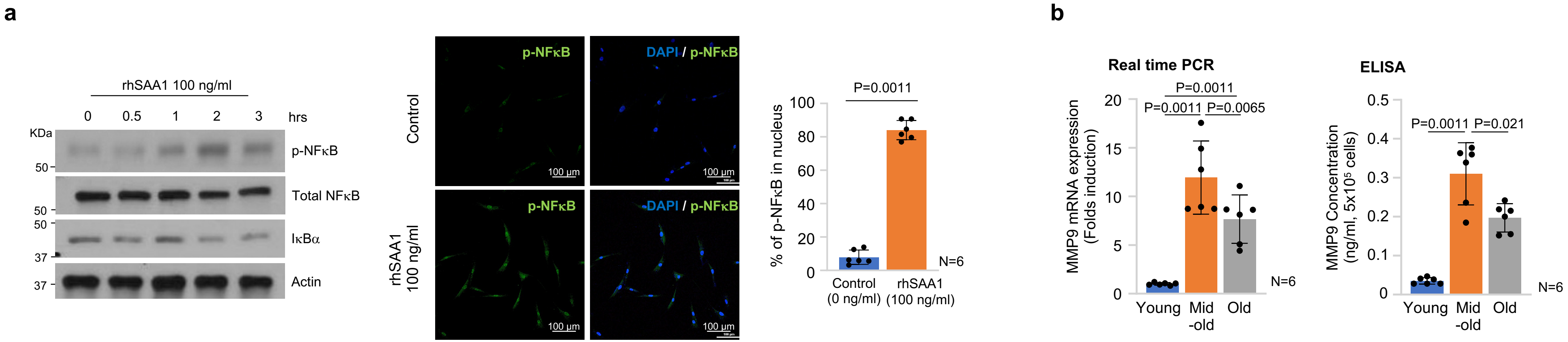

Supplementary Figure 10. NFκB signaling activation by SAA1 and the expression of MMP9 in young, mid-old, and old cells. a Young cells were treated with 100 ng/ml rhSAA1 for the indicated times, and immunoblotting was performed to assess activation of NFκB signaling (left panel). IF staining results for phosphorylated NFκB (p-NFκB) in fibroblasts treated with 100 ng/ml rhSAA1 for 30 mins are shown (right panel). The nuclear p-NFκB positive cells were quantified and are presented as bar graph (right). The *p* value was calculated using one-tailed Mann-Whitney U test. **b** MMP9 expression level was examined by real-time PCR (left panel) and ELISA (right panel) in young, mid-old, and old fibroblasts. The *p* values were calculated using the one-tailed Mann-Whitney U test. All bar graphs are presented as mean ± SD.

Supplementary Figure 11

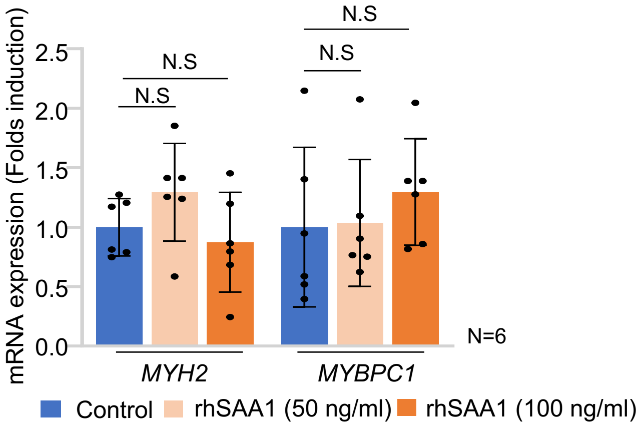

Supplementary Figure 11. The effect of SAA1 on the expression of muscle contraction-related genes in PASMCs. PASMCs were treated with rhSAA1 at the indicated concentrations (50 and 100 ng/ml) for 24 hrs. Muscle contraction-related genes' expression (*MYH2; Myosin heavy chain 2* and *MYBPC1; Myosin binding protein C1*) was determined using real-time PCR. The *p* values were calculated using the one-tailed Mann-Whitney U test. Data are presented mean ± SD.

Supplementary Figure 12

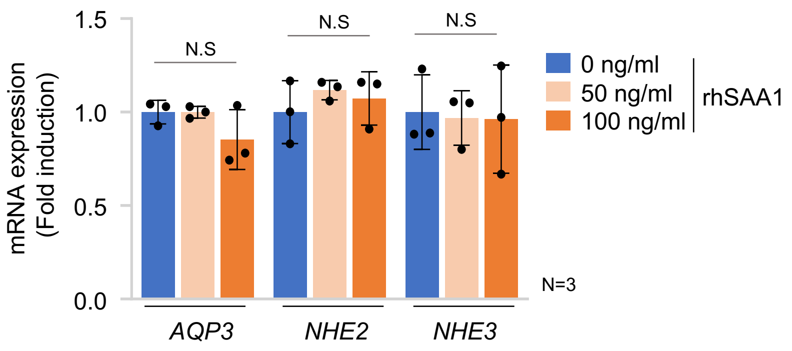

Supplementary Figure 12. The effect of SAA1 on the *AQP3, NHE2, or NHE3* expression in colonic epithelial cells. HCoEpiC colon epithelial cells was treated with rhSAA1 at indicated concentration for 48 hrs, and the expression of functional markers (*AQP3*, *NHE2,* and *NHE3*) was measured by real-time PCR. The *p* values were calculated using the one-tailed Mann-Whitney U test. Data are presented in mean ± SD.

Supplementary Figure 13

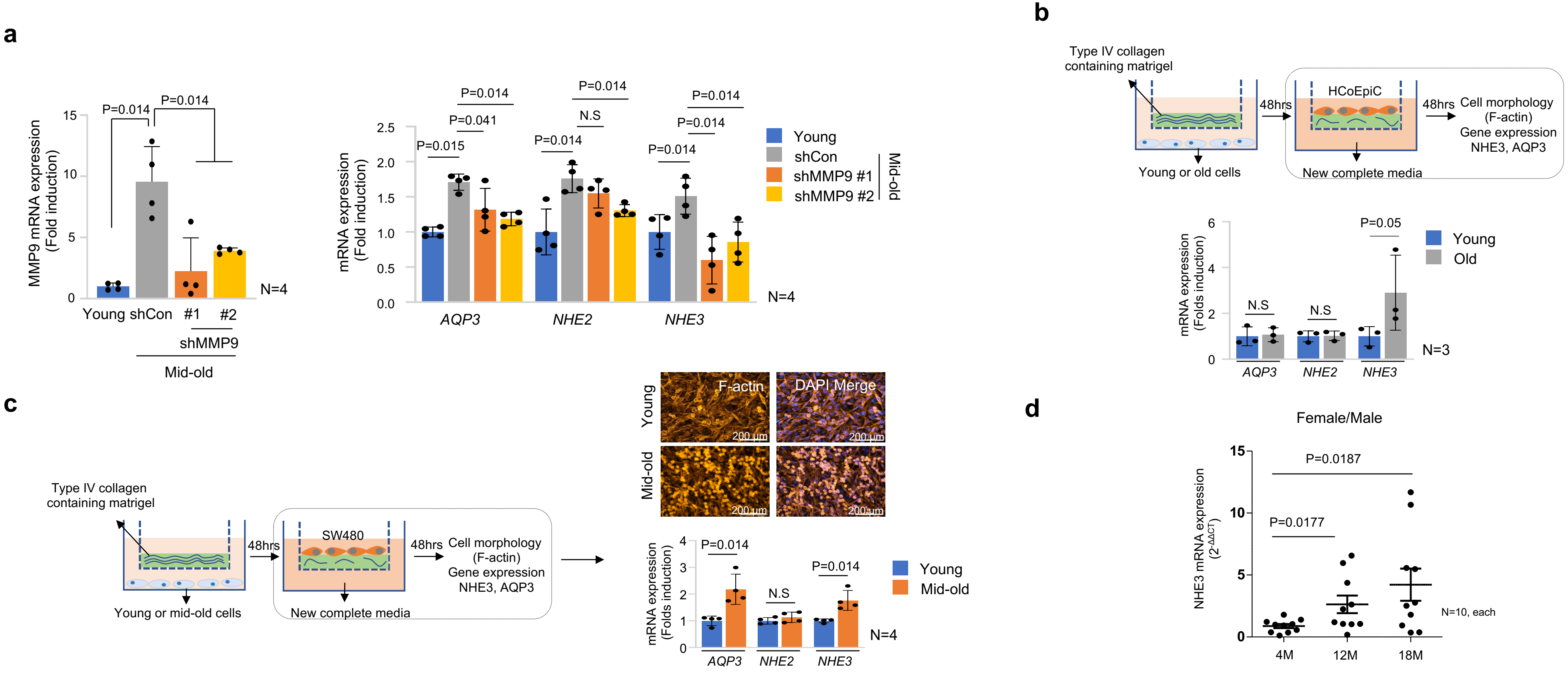

Supplementary Figure 13. The effect of the degradation of the BM by mid-old cells on epithelial cell's function. a Mid-old cells were infected with shMMP9-harboring lentivirus (left panel) and then co-cultured with HCoEpiC colon epithelial cells. After 2 days, the mRNA expression of *AQP3, NHE2*, and *NHE3* was analyzed using real-time PCR (right panel). **b** HCoEpic colonic epithelial cells were co-cultured with young or old fibroblasts. After 2 days, the mRNA expression of *AQP3, NHE2*, and *NHE3* was analyzed using real-time PCR. **c** Mid-old cells were co-cultured with SW480 cells. After 2 days, the mRNA expression of *AQP3, NHE2*, and *NHE3* was analyzed using real-time PCR. **d** The expression of *NHE3* in mouse colon tissue was examined. Total RNA was isolated from colon tissue obtained from male and female mice at different ages (4 months, 12 months, and 18 months), and real-time PCR was performed to measure *NHE3* expression. The *p* values in **a-d** were calculated using the one-tailed Mann-Whitney U test. Data in **a** are presented in mean + SD. Data in **b-d** are presented in mean ± SD.

Supplementary Figure 14

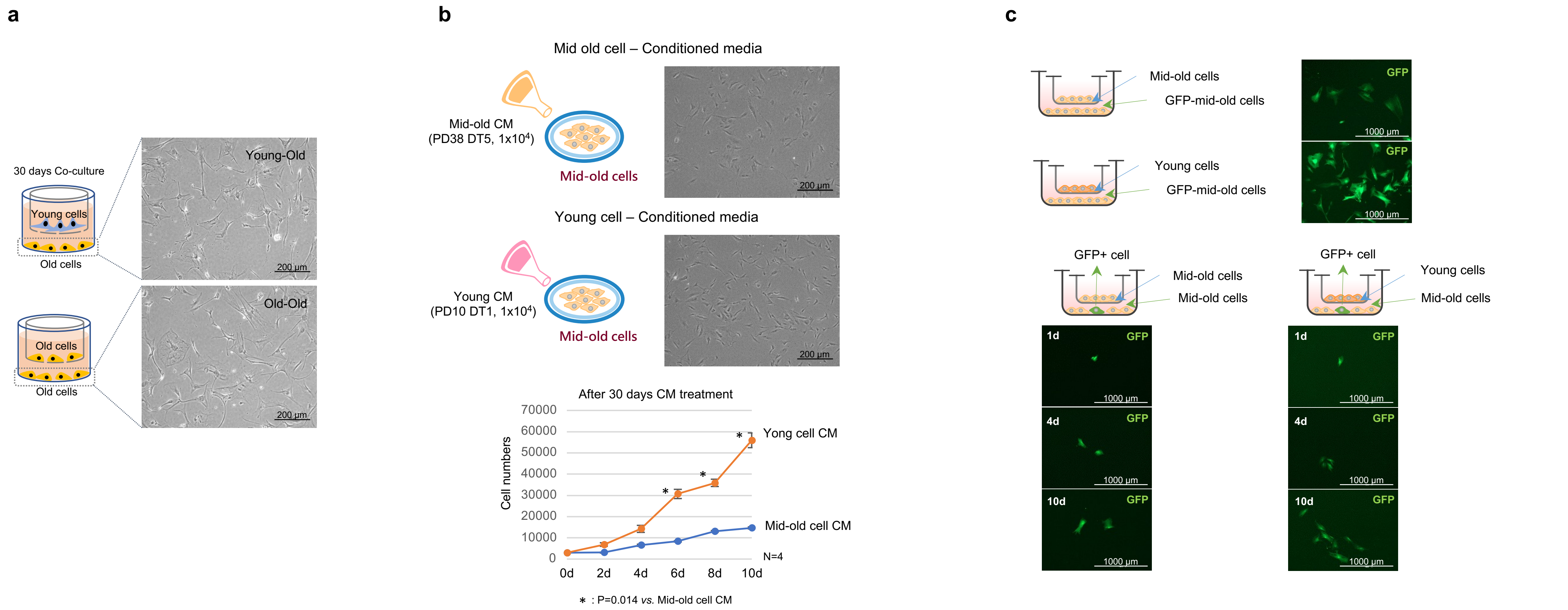

Supplementary Figure 14. The effect of young cell-driven factors on mid-old cells. a Old fibroblasts were co-cultured with old or young fibroblasts for 30 days. Representative images of co-cultured old cells are shown. **b** Mid-old cells were subjected to a treatment where conditioned medium (CM) extracted from either mid-old or young fibroblasts was applied for a duration of 30 days. The CM was changed every 3 days during the treatment. At the experimental endpoint, cell morphology was analyzed (upper panel). After the 30-day treatment with CM, the cell growth rate was assessed every 2 days for a period of 10 days by counting the number of cells (lower panel). The *p* values were calculated using the one-tailed Mann-Whitney U test. Data are presented in mean ± SD. **c** GFP-expressing mid-old cells (DT5) were co-cultured with either young (DT1) or mid-old cells (DT5) for a duration of 30 days. At the endpoint, GFP fluorescence was analyzed in the mid-old cells (upper panel). Individual mid-old cells were labeled with GFP and tracked for 30 days in the co-culture system.

Supplementary Figure 15

a

Metabolites from young cell conditioned media

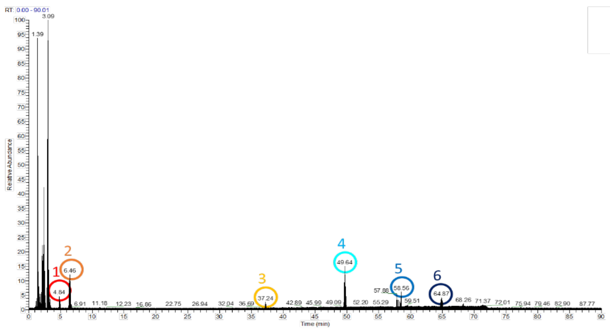

1	Methocarbamol	C11 H15 N O5
1	Thian-4-one 4-[6-(trifluoromethyl)-2-pyridyl]hydrazone	C11 H12 F3 N3 S
2	2-methyl-2,3,4,5-tetrahydro-1,5-benzoxazepin-4-one	C10 H11 N O2
2	1,5-Naphthalenediamine	C10 H10 N2
2	DL-Tryptophan	C11 H12 N2 O2
2	XY5850570	C6 H10 Cl N5
3	Folic acid	C19 H19 N7 O6
4	2-[2-(2,3-dihydro-1,4-benzodioxin-6-yl)-1,3-thiazol-4-yl]-1,3-benzothiazole	C18 H12 N2 O2 S2
5	Cyprodenate	C13 H25 N O2
5	L-lanthionine	C6 H12 N2 O4 S
5	cis-1,2-dihydroxy-1,2-dihydrodibenzothiophene	C12 H10 O2 S
5	4253	C12 H22 O2
5	(R)-3-Hydroxy myristic acid	C14 H28 O3
6	prostaglandin G2 2-glyceryl ester	C23 H38 O8
6	1-Stearoylglycerol	C21 H42 O4
6	1-Linoleoyl-2-Hydroxy-sn-glycero-3-PC	C26 H50 N O7 P

b

Lipids from young cell conditioned media

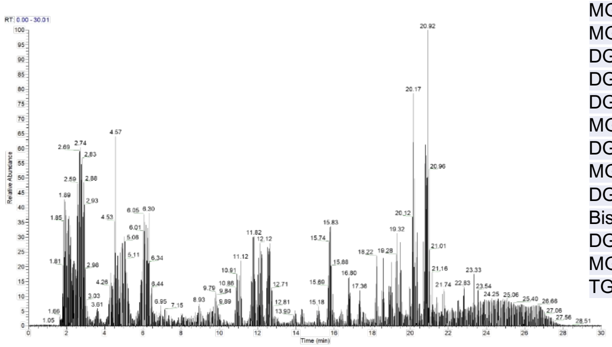

WE(3:0_18:0)+H	WE	TG(12:0e_6:0_6:0)+NH4	TG	TG(12:0e_6:0_16:0)+NH4	TG	SiE(24:1)+NH4	SiE	TG(20:2e_11:4_24:2)+Na	TG
MG(18:2)+H	MG	TG(6:0_6:0_12:1)+NH4	TG	DG(18:0_16:0)+NH4	DG	PC(18:1_18:2)+H	PC	TG(18:1_18:1_18:1)+Na	TG
MG(18:0)+H	MG	MG(26:0)+NH4	MG	DG(20:0_16:0)+NH4	DG	PC(18:0_18:2)+H	PC	TG(26:1_6:0_23:1)+NH4	TG
DG(6:0_12:0)+H	DG	DG(15:0_10:2)+Na	DG	DG(18:0_18:0)+NH4	DG	DG(33:1_14:0)+NH4	DG	TG(18:0_16:1_22:6)+NH4	TG
DG(8:0_10:0)+H	DG	TG(12:0e_6:0_8:0)+NH4	TG	TG(18:0_8:0_10:3)+NH4	TG	MePC(17:1_18:2)+Na	MePC	TG(27:1_6:0_24:2)+NH4	TG
DG(9:0_9:0)+H	DG	BisMePA(4:0_15:0)+NH4	BisMePA	TG(11:0_10:0_15:0)+NH4	TG	TG(15:0_16:0_16:1)+NH4	TG	TG(29:1_6:0_24:2)+NH4	TG
MG(19:0)+H	MG	DG(16:2e_14:0)+H	DG	DG(20:1e_18:1)+Na	DG	PC(18:0_20:4)+H	PC	TG(30:0_16:1_22:4)+NH4	TG
DG(11:0_6:0)+NH4	DG	TG(12:0e_8:0_8:0)+NH4	TG	SiE(18:0)+NH4	SiE	MePC(19:1_18:2)+Na	MePC		
MG(18:0)+NH4	MG	CmE(8:0)+NH4	CmE	SiE(20:5)+H	SiE	PC(20:0_20:4)+H	PC		
DG(6:0_13:0)+NH4	DG	DG(20:0_10:3)+NH4	DG	CmE(20:5)+NH4	CmE	TG(16:0_16:1_17:1)+Na	TG		
BisMePA(6:0_8:0)+NH4	BisMePA	PE(8:0e_16:0)+Na	PE	StE(20:5)+NH4	StE	TG(18:0_16:1_17:1)+NH4	TG		
DG(18:3e_6:0)+Na	DG	DG(28:0_6:0)+H	DG	TG(16:0_6:0_20:3)+NH4	TG	TG(18:0_10:1_24:1)+NH4	TG		
MG(24:0)+NH4	MG	TG(4:0_12:0_16:0)+NH4	TG	StE(22:4)+NH4	StE	TG(20:1_10:1_22:0)+NH4	TG		
TG(12:1e_6:0_6:0)+NH4	TG	DG(28:0_6:0)+NH4	DG	SiE(22:1)+NH4	SiE	TG(14:0_14:0_24:0)+NH4	TG		

Supplementary Figure 15. Analysis of metabolites and lipids in CM from young cells. CM from young and mid-old (n=3, each) are analyzed using LC-MS. CM from mid-old was used as a control. The raw and processed file can be found in the online repository with accession code, ST002960 and ST002961. Representative images of **a** metabolites and **b** lipids in conditioned medium obtained from young cell measured using an LC-MS chromatogram. WE: Fatty esters wax monoexters 4, MG: Monoacylglycerols, DG: Diacylglycerols, TG; Triacylglycerols, BisMePA: bis-methyl phosphatidic acid, CmE: Cholesteryl methyl ester, PE: Phosphatidylethanolamines, SiE: Sitosterol ester, StE: Stigmasterol ester, MePC: Methyl phosphatidylcholine, PC: Phosphatidylcholine.

Supplementary Figure 16

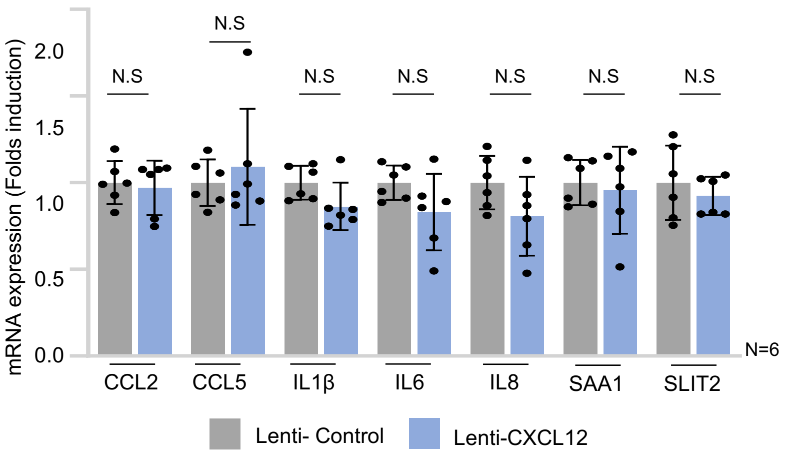

Supplementary Figure 16. The effect of CXCL12 overexpression on the SASP expression of mid-old cells. Mid-old fibroblasts were infected with a lentivirus harboring CXCL12 overexpression vector for 1 week, and the expression of SASP was assessed using real-time PCR. The *p* values were calculated using the one-tailed Mann-Whitney U test. Data are presented in mean ± SD.

Supplementary Figure 17

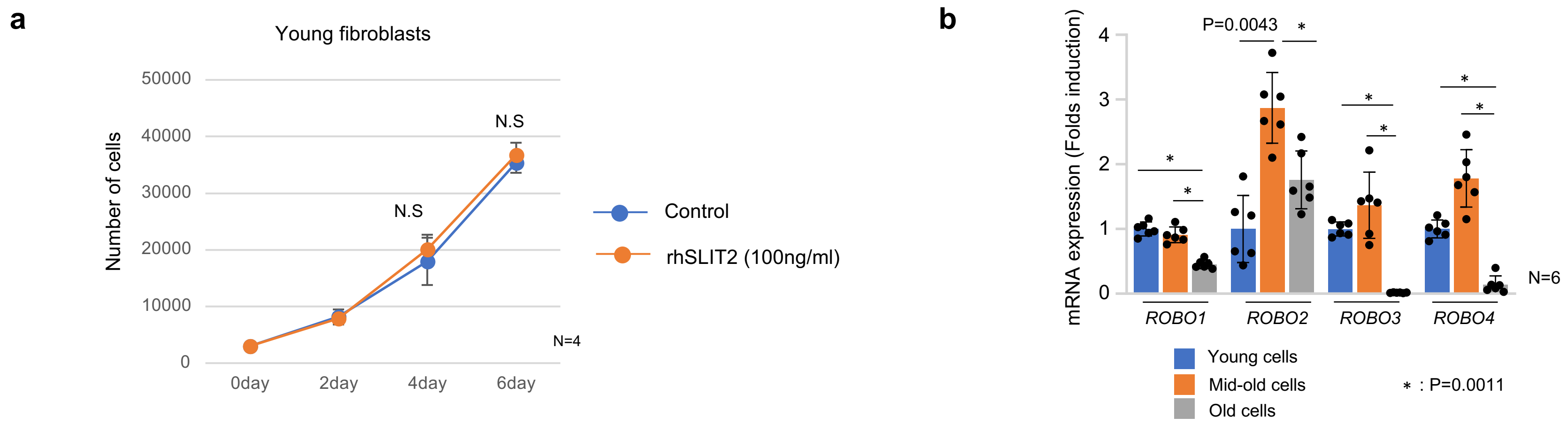

Supplementary Figure 17. The response of young cells to rhSLIT2 and the expression of ROBO receptors in young, mid-old, and old cells. **a** Young fibroblasts were treated with rhSLIT2 (100ng/ml) for 6 days. Cell proliferation was analyzed while treating cells with rhSLIT2 for 6 days. The cells were treated with rhSLIT2 every 2 days with media changes. **b** ROBO receptors' expression was analyzed in young, mid-old, and old cells using real-time PCR. The *p* values in **a-b** were calculated using the one-tailed Mann-Whitney U test. Data are presented in mean ± SD.

Supplementary Figure 18

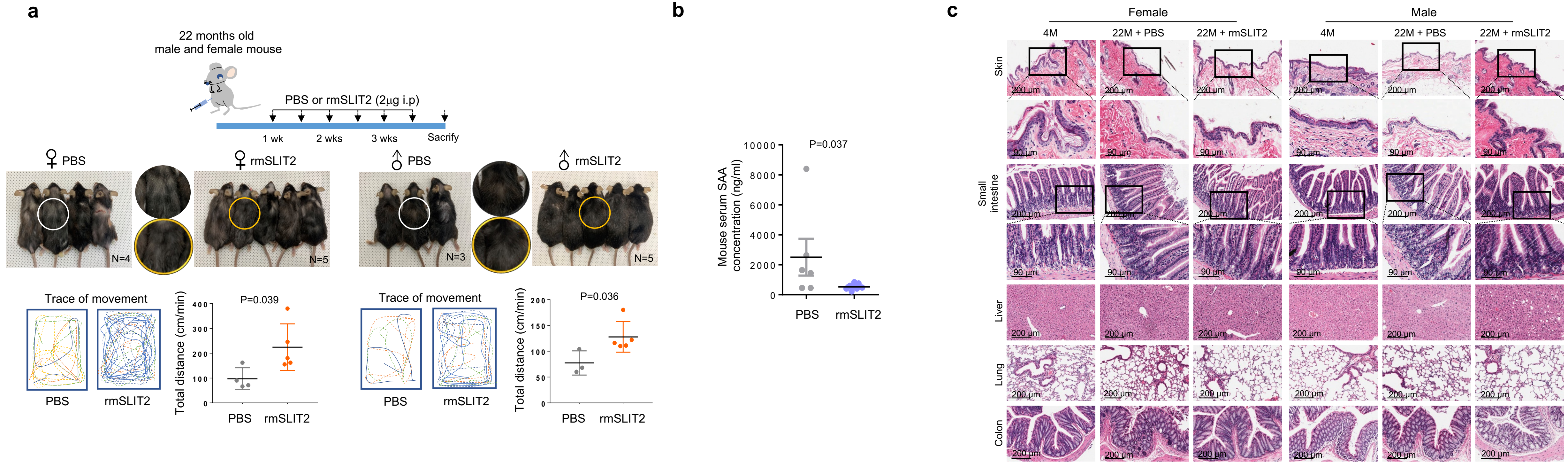

Supplementary Figure 18. The effect of rmSLIT2 administration on the aged mice activity. In the study, 22-month-old male and female mice (n = 4; PBS, n = 5; rmSLIT2 and n = 4; PBS, n = 5; rmSLIT2, respectively) were intraperitoneally injected with PBS (control) or rmSLIT2 (2 μg/mouse) twice weekly for a duration of 3 weeks. One male mouse that received treatment with PBS died during the course of the experiment. **a** To assess the effectiveness of rmSLIT2, the movement activity of the mice in their cages was evaluated at the endpoint of the experiment. The movement activity was measured as the total distance covered per minute. **b** Serum SAA (serum amyloid A) levels were measured in mice treated with PBS and rmSLIT2. **c** Histological evaluation was performed in mice treated with PBS and rmSLIT2 to assess any differences in tissue structure or characteristics. The *p* values in **a**-**b** were calculated using the one-tailed Mann-Whitney U test. Data are presented in mean ± SD.

Supplementary Figure 19

	PBS					rmSLIT2					Mean ± SD		*p*-value
Animal No.	1	2	3	4	5	1	2	3	4	5	PBS	rmSLIT2	PBS *vs.* rmSLIT2
pH	7.318	7.252	7.207	7.267	7.26	7.142	7.41	7.199	7.224	7.315	7.3 ± 0.04	7.3 ± 0.10	0.69
pCO_2 (mmHg)	38	46.3	44.3	48.9	51.9	36.5	30.7	63.6	50.1	40.6	45.9 ± 5.25	44.3 ± 12.9	0.69
Na^+ (mmol/L)	148	116	138	125	134	104	147	145	148	148	132.2 ± 12.3	138.4 ± 19.2	0.41
K^+ (mmol/L)	4.6	4.3	7	7.4	5.8	5.7	4	5.4	5.6	4.6	5.8 ± 1.38	5.1 ± 0.73	0.33
Ca^{2+} (mmol/L)	1.24	0.64	0.89	0.82	0.97	0.48	1.2	1.29	1.13	1.27	0.9 ± 0.22	1.1 ± 0.34	0.31
HCO_3^- (mmol/L)	20.1	19	17.1	20.5	21.3	12.8	21.4	21.1	19.6	21	19.6 ± 1.62	19.2 ± 3.63	0.69
Anion gap	19	19	22	22	17	18.3	17	22	19	15	19.8 ± 2.17	18.3 ± 2.58	0.37

Supplementary Figure 19. Blood analysis in PBS and rmSLIT2 treated mice. In the study, 23-month-old male mice were intraperitoneally injected twice weekly for a period of 5 weeks with either PBS (control, n = 5) or rmSLIT2 (2 µg/mouse, n = 5). Blood samples were collected from the jugular vein and immediately analyzed. The analysis included parameters such as pH, pCO_2, and electrolyte levels. The *p* values are obtained from two-tailed Mann-Whitney U test.

Supplementary Figure 20

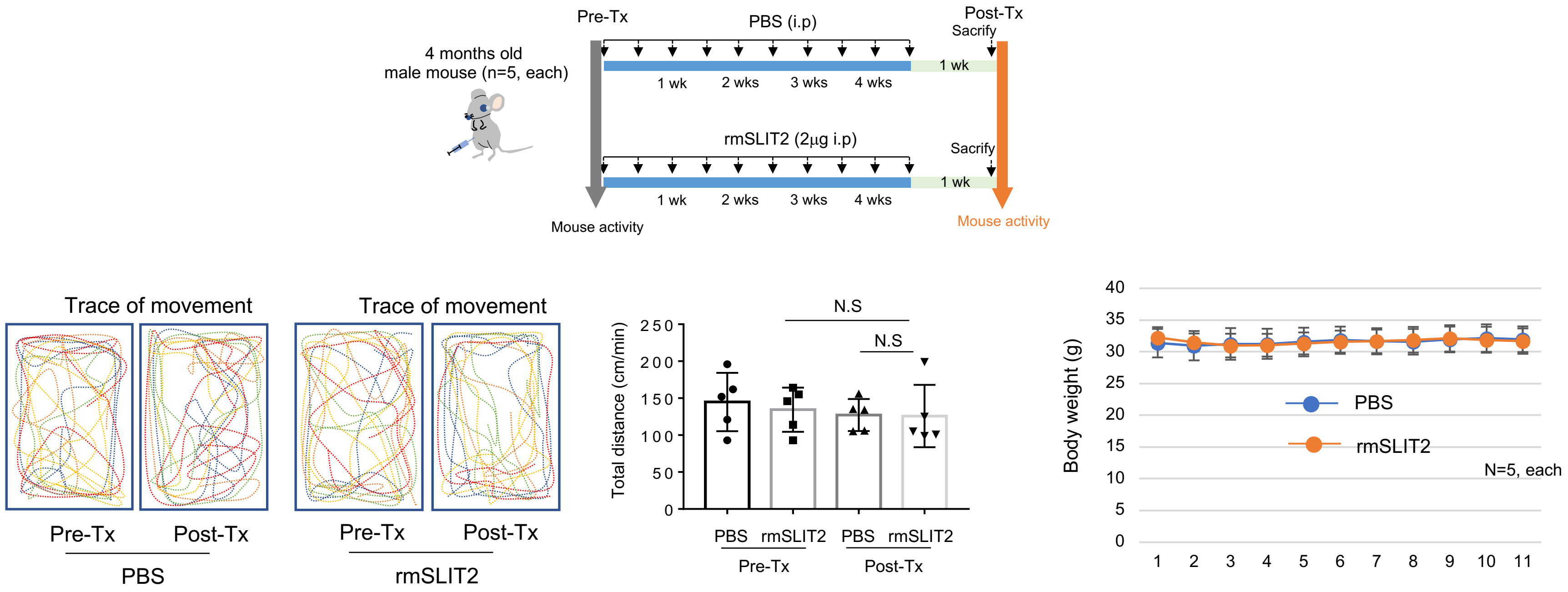

Supplementary Figure 20. Assessment of the activity and body weight after treating rmSLIT2 in young mice. 4-month-old male mice (n = 5 for PBS, n = 5 for rmSLIT2) were intraperitoneally injected twice weekly with either PBS (control) or rmSLIT2 (2 μg/mouse) for 5 weeks. Movement activity in the cage was manually recorded and plotted (left panel). Total distance was calculated, and statistical analysis is shown in the middle panel. The *p* value was calculated using one-tailed Mann-Whitney U test. Data are presented in mean ± SD. The right panel displays body weight changes during treatment with PBS or rmSLIT2, respectively.

Supplementary Figure 21

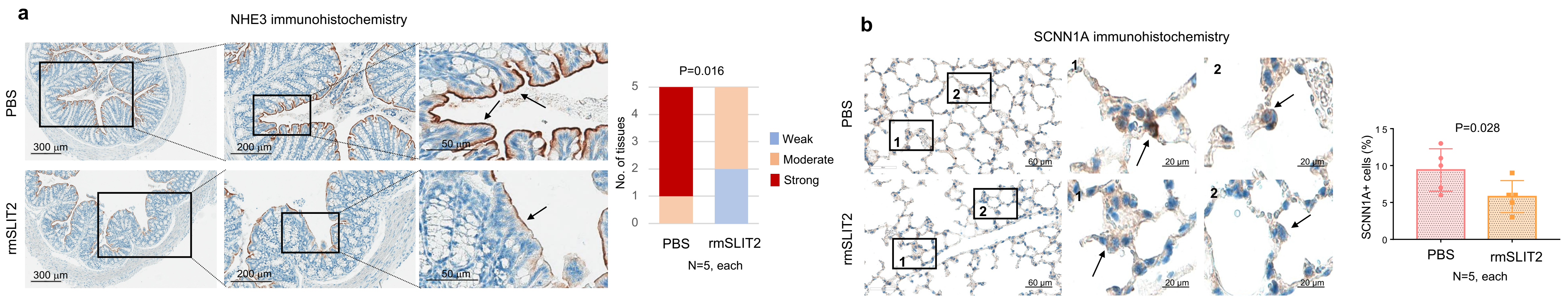

Supplementary Figure 21. NHE3 and SCNN1A protein expression in rmSLIT2 treated mice. NHE3 and SCNN1A protein expression in colon and lung of PBS (control) or rmSLIT2 (2 μg/mouse) treated mouse was evaluated by IHC analysis (n = 5; PBS, n = 5; rmSLIT2, respectively). **a** NHE3 was stained in the apical region of the colon epithelium. Each protein expression status was evaluated as weak, moderate, and strong, respectively. The *p* value is obtained from Chi-square test. **b** SCNN1A expression in alveolar cell was presented as the percentage of positive cells. The *p* value was determined using the one-tailed Mann-Whitney U test. Data are presented in mean ± SD.

Supplementary Figure 22

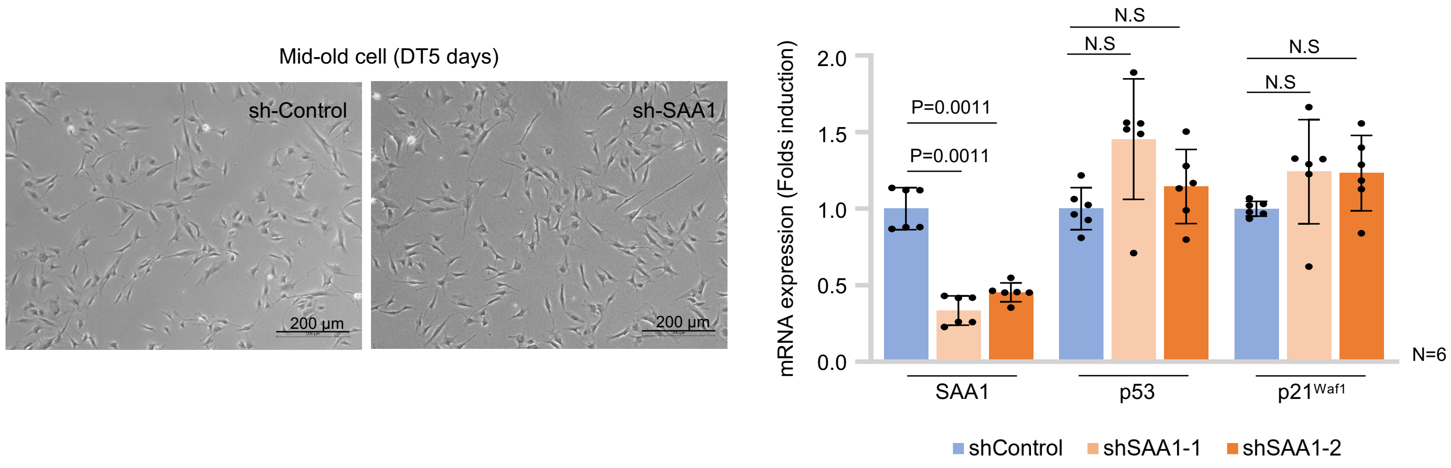

Supplementary Figure 22. The effect of SAA1 downregulation on proliferation of mid-old cells. Mid-old fibroblasts were infected with shSAA1 for 24hrs, and the culture media was changed to fresh media. Cells were harvested after 56 hrs. Following the infection, the mRNA expression of SAA1, p53, $p21^{Waf1}$ was measured, and the cell morphology was assessed. The *p* values were calculated using the one-tailed Mann-Whitney U test. Data are presented in mean ± SD.

Supplementary Figure 23

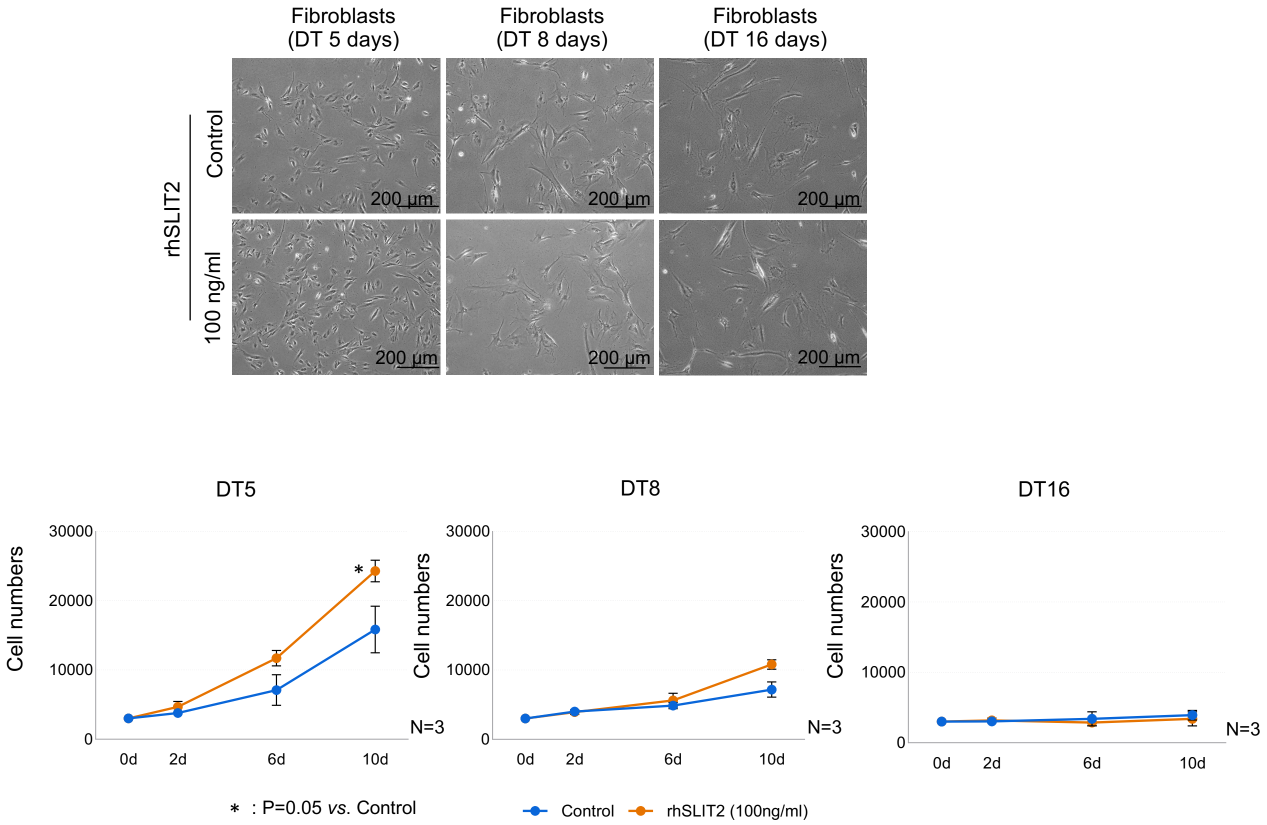

Supplementary Figure 23. SLIT2 specifically stimulates the growth of mid-old cells. The fibroblasts were treated with rhSLIT2 (100 ng/ml) at different time points of their replicative senescence, specifically at DT5, DT8, and DT16, for a duration of 10 days. The morphology of the cells (upper panel) and their growth rate (lower panel) were analyzed. The *p* values were calculated using the one-tailed Mann-Whitney U test. Data are presented in mean ± SD.
