# Supplementary material for: Mid-old cells are a potential target for anti-aging interventions in the elderly
Source: Nat Commun. 2023 Nov 22;14:7619. doi: 10.1038/s41467-023-43491-w (PMC10665435; doi:10.1038/s41467-023-43491-w)
Supplement: Supplementary file 3 — Description of Additional Supplementary Files [file 41467_2023_43491_MOESM3_ESM.pdf]

Description of Additional Supplementary Files:

Supplementary Data 1: Gene expression changes with repetitive replication: genes decreasing, increasing, and highest in mid-old cells.

Supplementary Data 2: GSEA results of functional gene sets of the fibroblast.

Supplementary Movie 1: Representative Erk1/2-KTR-mClover translocation patterns in young, mid-old and old cells. Each type of cell was starved for 24 hrs using serum-free media before the experiment. Fluorescent images were acquired every 15 sec for 30 mins with a 100 msec exposure after serum (20%) stimulation (three independent experiments with n=4 for young cells, n=6 for mid-old cells, and n=3 for old cells). The green channel represents Erk1/2-KTR-mClover, and the blue channel represents the nucleus stained with Hoechst.

Supplementary Movie 2: Activity of old-aged C57BL/6J mice after administration of rmSLIT2. PBS or rmSLIT2 protein was injected intraperitoneally to 22-month-old C57BL/6J mice six times for three weeks (n=4 PBS and n=5 rmSLIT2-treated, male and female each). Representative video of each group was displayed.

Supplementary Movie 3: Activity of old-aged C57BL/6N mice after administration of rmSLIT2. PBS or rmSLIT2 protein was injected intraperitoneally to 23-month-old male C57BL/6N mice ten times for five weeks (n=5, each). Representative video of each group was displayed.

Supplementary Movie 4: Activity of young-aged C57BL/6N mice after administration of rmSLIT2. PBS or rmSLIT2 protein was injected intraperitoneally to 4-month-old male C57BL/6N mice ten times for five weeks (n=5, each). Representative video of each group was displayed.
