# Supplementary material for: Mid-old cells are a potential target for anti-aging interventions in the elderly
Source: Nat Commun. 2023 Nov 22;14:7619. doi: 10.1038/s41467-023-43491-w (PMC10665435; doi:10.1038/s41467-023-43491-w)
Supplement: Supplementary file 10 — Reporting Summary [file 41467_2023_43491_MOESM10_ESM.pdf]

nature portfolio

Corresponding author(s): Jang-Hee Kim and Tae Jun Park

Last updated by author(s): Nov 6, 2023

Reporting Summary

Nature Portfolio wishes to improve the reproducibility of the work that we publish. This form provides structure for consistency and transparency in reporting. For further information on Nature Portfolio policies, see our Editorial Policies and the Editorial Policy Checklist.

Statistics

For all statistical analyses, confirm that the following items are present in the figure legend, table legend, main text, or Methods section.

n/a	Confirmed	
☐	☒	The exact sample size (*n*) for each experimental group/condition, given as a discrete number and unit of measurement
☐	☒	A statement on whether measurements were taken from distinct samples or whether the same sample was measured repeatedly
☐	☒	The statistical test(s) used AND whether they are one- or two-sided<br>*Only common tests should be described solely by name; describe more complex techniques in the Methods section.*
☐	☒	A description of all covariates tested
☐	☒	A description of any assumptions or corrections, such as tests of normality and adjustment for multiple comparisons
☐	☒	A full description of the statistical parameters including central tendency (e.g. means) or other basic estimates (e.g. regression coefficient) AND variation (e.g. standard deviation) or associated estimates of uncertainty (e.g. confidence intervals)
☐	☒	For null hypothesis testing, the test statistic (e.g. *F*, *t*, *r*) with confidence intervals, effect sizes, degrees of freedom and *P* value noted<br>*Give P values as exact values whenever suitable.*
☒	☐	For Bayesian analysis, information on the choice of priors and Markov chain Monte Carlo settings
☒	☐	For hierarchical and complex designs, identification of the appropriate level for tests and full reporting of outcomes
☒	☐	Estimates of effect sizes (e.g. Cohen's *d*, Pearson's *r*), indicating how they were calculated

Our web collection on statistics for biologists contains articles on many of the points above.

Software and code

Policy information about availability of computer code

Data collection	Zeiss microscope: Zen 2011 program<br>Leica microscope: Leica Application Suite (LAS) software<br>LeiCa Aperio Scanscope: Aperio imageScope software
Data analysis	Microsoft Excel and Prism 7 program were used for statistical analysis.<br>Image processing and quantification were carried out with Zen program and DP-Manager software.<br>R studio was used to process bulk RNA-sequencing and GSEA.<br>MATLAB was used forive cell imaging analysis.

For manuscripts utilizing custom algorithms or software that are central to the research but not yet described in published literature, software must be made available to editors and reviewers. We strongly encourage code deposition in a community repository (e.g. GitHub). See the Nature Portfolio guidelines for submitting code & software for further information.

Data

Policy information about availability of data

All manuscripts must include a data availability statement. This statement should provide the following information, where applicable:

- Accession codes, unique identifiers, or web links for publicly available datasets
- A description of any restrictions on data availability
- For clinical datasets or third party data, please ensure that the statement adheres to our policy

The data presented in this study are publicly available in NCBI's Gene expression Omnibus (GEO) and Metabolomics Workbench under the accession codes:
GSE232425 [https://www.ncbi.nlm.nih.gov/geo/query/acc.cgi?acc=GSE232425],
GSE232427 [https://www.ncbi.nlm.nih.gov/geo/query/acc.cgi?acc=GSE232427],
GSE201457 [https://www.ncbi.nlm.nih.gov/geo/query/acc.cgi?acc=GSE201457],
GSE41714 (cDNA microarray) [https://www.ncbi.nlm.nih.gov/geo/query/acc.cgi?acc=GSE41714], GSE178341 (scRNA-seq) [https://www.ncbi.nlm.nih.gov/geo/query/acc.cgi?acc=GSE178341],
ST002960 (metabolomics) [http://dev.metabolomicsworkbench.org:22222/data/DRCCMetadata.php?Mode=Study&StudyID=ST002960&Access=ZirF6602], and
ST002961 (lipidomics) [http://dev.metabolomicsworkbench.org:22222/data/DRCCMetadata.php?Mode=Study&StudyID=ST002961&Access=IrhC2910]. Source data are provided with this paper.

Human research participants

Policy information about studies involving human research participants and Sex and Gender in Research.

Reporting on sex and gender	Patients' sex, age and diagnosis information was collected from Medical data from Ajou University Hospital.
Population characteristics	Paraffin block of patients who got surgical resection in Ajou University Hospital from 2018 to 2020 were collected.
Recruitment	To exclude possibility potential contamination with tumor tissue, paraffin blocks which have normal tissue 5-12 cm away from the pathologic regions were exclusively selected by pathologists. Paraffin blocks with normal tissue less than 5-12cm away from the pathologic regions were previously excluded.
Ethics oversight	Ajou University Medical Center

Note that full information on the approval of the study protocol must also be provided in the manuscript.

Field-specific reporting

Please select the one below that is the best fit for your research. If you are not sure, read the appropriate sections before making your selection.

☒ Life sciences ☐ Behavioural & social sciences ☐ Ecological, evolutionary & environmental sciences

For a reference copy of the document with all sections, see nature.com/documents/nr-reporting-summary-flat.pdf

Life sciences study design

All studies must disclose on these points even when the disclosure is negative.

Sample size	Sample size were not predetermine but counting for cell number and positive signal cell number were performed three independent replicate experiments
Data exclusions	N/A
Replication	All data were analyzed in three independent experiments
Randomization	For calculating cell number per filed the imaging were selected randomly
Blinding	Investigators were not blinded. Quantification of counting cell number and positive signal cell number applied equally conditions and replicates experiment.

Reporting for specific materials, systems and methods

We require information from authors about some types of materials, experimental systems and methods used in many studies. Here, indicate whether each material, system or method listed is relevant to your study. If you are not sure if a list item applies to your research, read the appropriate section before selecting a response.

Materials & experimental systems

n/a	Involved in the study	
☐	☒	Antibodies
☐	☒	Eukaryotic cell lines
☒	☐	Palaeontology and archaeology
☐	☒	Animals and other organisms
☒	☐	Clinical data
☒	☐	Dual use research of concern

Methods

n/a	Involved in the study	
☒	☐	ChIP-seq
☒	☐	Flow cytometry
☒	☐	MRI-based neuroimaging

Antibodies

Antibodies used

1. Immunohistochemical staining:
human p16INK4A, predilution (805-4713, Roche); human Ki67, 1:3000 (M7240, Dako); human H3K9me3, 1:500 (ab176916, Abcam); human p21Waf1, 1:300 (2990-1, Epitomics); human IL1β, 1:100 (ab9722, Abcam); human SAA1, 1:75 (MAB30191, R&D System); human SLIT2, 1:80 (HPA023088, Atlas Antibodies); human type IV Collagen, 1:300 (ab6586, Abcam); human MMP9, 1:100 (GTX100458, Genetex Inc.); human CXCL12, 1:100 (MAB350, R&D System); human CCL5, 1:200 (MAB278, R&D System); mouse p16INK4A, 1:300. (ab189034, Abcam); mouse Ki67, 1:200 (ab16667, Abcam); mouse SAA1/2, 1:1000 (ab199030, Abcam); mouse SLIT2, 1:200 (PA5-31133, Invitrogen); human/mouse NHE3, 1:500 (NBP1-82574, Novus Biologicals); human/mouse SCNN1A, 1:100 (ab272878, Abcam); mouse SOX2, 1:100 (ab92494, Abcam).
2. Multiplex IHC staining:
p16INK4A (predilution, 805-4713, Roche)/CD3(1:150, ab135372, Abcam), p16INK4A (predilution, 805-4713, Roche)/CD19 (1:100, ab227688, Abcam), and p16INK4A (predilution, 805-4713, Roche)/CD68 (1:2,500, NBP2-48923, Novus Biologicals) was performed using BOND Polymer Refine Detection kit (DS9800, Leica Biosystems, Wetzlar, Germany) and green chromogen kit (DC9913, Leica Biosystems) in the BOND-III fully automated IHC staining system (22.2201, Leica Biosystems).
3. Immunofluorscence staining of FFPE tissue sections:
SAA1, 1:50 (MAB30191, R&D Systems); p21Waf1, 1:100 (ab109520, Abcam); IL1β, 1:100 (ab9722, Abcam); SLIT2, 1:200 (PA5-31133, Invitrogen); p16INK4A, predilution (805-4713, Roche); Vimentin, 1:400 (ab92547, Abcam); Vimentin, 1:100 (AF2105, R&D Systems); Smoothelin, 1:100 (OMA1-06020, Invitrogen); Smoothelin, 1:500 (NBP2-37931, Novus Biologicals); E-cadherin, 1:200 (610181, Becton Dickinson); E-cadherin, 1:100 (ab15148, Abcam); CDX2, 1:100, (235R-15, Cell Margue, Rocklin, CA); Laminin antibody, 1:500 (ab11575, Abcam); Alexa Fluor 488, 1:300 (ab150113, Abcam); Alexa Flour 488, 1:300 (A-11055, Invitrogen); Alexa Flour 488, 1:300 (A-21206, Invitrogen); Alexa Flour 405, 1:300 (A48255, Invitrogen); Alexa Fluor 594, 1:300 (ab150080, Abcam); Alexa Fluor 594, 1:300 (ab150116, Abcam).
4. Immunofluorscence staining of cells:
Ki67, 1:500 (M7240, Dako); p-Erk1/2, 1:100 (9101, Cell Signaling Technology); p-NFκB, 1:100 (3033, Cell Signaling Technology); p21Waf1, 1:100 (ab109520, Abcam); p16INK4A, 1:300 (ab189034, Abcam); TRITC- phalloidin, 1:500 (P1951, Sigma); Alexa Fluor 488, 1:600 (A-21206, Thermo Fisher Scientific); Alexa Fluor 555, 1:600 (A-31572, Thermo Fisher Scientific).
5. Immunoblotting:
p16INK4A, 1:1000 (LS-B1347, LSbio, Seattle, WA); p21Waf1, 1:1000 (ab109520, Abcam); p53, 1:1000 (MA5-12557, Invitrogen); p-Erk1/2, 1:1000 (9101, Cell Signaling Technology); Total Erk1/2, 1:1000 (9107, Cell Signaling Technology); MDM2, 1:1000 (ab16895, Abcam); Actin, 1:3000 (Abc-2004, Abclon); p-NFκB, 1:1000 (3033, Cell Signaling Technology); Total NFκB, 1:1000 (8242, Cell Signaling Technology); IκBα, 1:1000 (4814, Cell Signaling Technology); p-Pyk2, 1:1000 (3291, Cell Signaling Technology); Total Pyk2, 1:1000 (3292, Cell Signaling Technology); SOX2, 1:500 (ab92494, Abcam); OCT4, 1:1000 (ab19857, Abcam); MMP9, 1:1000 (GTX100458, Genetex Inc.); Tubulin, 1:1000 (sc-32293, Santa Cruz Biotechnology inc.); GAPDH, 1:1000 (60004-1-lg, Proteintech); Type IV collagen, 1:1000 (ab6586, Abcam).

Validation

All validation statements were taken from suppliers website.

Eukaryotic cell lines

Policy information about cell lines and Sex and Gender in Research

Cell line source(s)

Primary normal human dermal fibroblasts, pulmonary artery smooth muscle cells (PASMCs), Human pulmonary alveolar epithelial cells (HPAEpiC), human primary colonic epithelial cells (HCoEpiC), SW480 cells and IMR90 cells were used in this study. The primary cell's personal infromation was not provide to protect privacy.

Authentication

Primary normal human dermal fibroblasts were isolated from foreskin. Primary pulmonary artery smooth muscle cells (PASMCs), SW480 and IMR90 cells were purchased from American Tissue Culture Collections (ATCC, Rockville, MD). Human pulmonary alveolar epithelial cells (HPAEpiC) and human primary colonic epithelial cells (HCoEpiC) were purchase from ScienCell Research Laboratories, Inc. (Carlsbad, CA) and Cell Biologics, Inc. (Chicago, IL), respectively.

Mycoplasma contamination

All cell lines were confirmed free of mycoplasma contamination by MycoLuor Mycoplasma Detection Kit (Invitrogen, #M7006)

Commonly misidentified lines
(See ICLAC register)

N/A

Animals and other research organisms

Policy information about studies involving animals; ARRIVE guidelines recommended for reporting animal research, and Sex and Gender in Research

Laboratory animals	Male and female C57BL/6J and male C57BL/6N mice
Wild animals	N/A
Reporting on sex	Male and female C57BL/6J mice (4-month-old and 12-month-old) were purchased from KBSI (Korea Basic Science Institute, Gwang-ju, Korea). We are consider male and female mice in this experiment. Male C57BL/6N mouse were purchased from ORIENT BIO (Seongnam, Korea).
Field-collected samples	Blood was immediately collected by intracardial puncture and then serum was collected by centrifuging samples. Colon, small intestine, stomach, lung, liver and skin were isolated and embedded in paraffin. The skeletal muscles from the hindlimb were dissected and embedded in an OCT compound.
Ethics oversight	All animal procedures were approved by the institutional animal research ethics committee at Ajou University Medical Center (approval number: 2020-0051).

Note that full information on the approval of the study protocol must also be provided in the manuscript.
